# The Potential Role of the Dipeptidyl Peptidase-4-Like Activity From the Gut Microbiota on the Host Health

**DOI:** 10.3389/fmicb.2018.01900

**Published:** 2018-08-22

**Authors:** Marta Olivares, Valentina Schüppel, Ahmed M. Hassan, Martin Beaumont, Audrey M. Neyrinck, Laure B. Bindels, Alfonso Benítez-Páez, Yolanda Sanz, Dirk Haller, Peter Holzer, Nathalie M. Delzenne

**Affiliations:** ^1^Metabolism and Nutrition Research Group, Louvain Drug Research Institute, Université catholique de Louvain, Brussels, Belgium; ^2^ZIEL Institute for Food and Health, Technical University of Munich, Freising-Weihenstephan, Germany; ^3^Chair of Nutrition and Immunology, Technical University of Munich, Freising-Weihenstephan, Germany; ^4^Research Unit of Translational Neurogastroenterology, Pharmacology Section, Otto Loewi Research Center, Medical University of Graz, Graz, Austria; ^5^Microbial Ecology, Nutrition and Health Research Unit, Institute of Agrochemistry and Food Technology, Spanish National Research Council (IATA-CSIC), Valencia, Spain

**Keywords:** DPP-4 activity, PepX activity, gut microbiota, microbiome, metabolism, behavior

## Abstract

The Dipeptidyl peptidase-4 (DPP-4) activity influences metabolic, behavioral and intestinal disorders through the cleavage of key hormones and peptides. Some studies describe the existence of human DPP-4 homologs in commensal bacteria, for instance in *Prevotella* or *Lactobacillus*. However, the role of the gut microbiota as a source of DPP-4-like activity has never been investigated. Through the comparison of the DPP-4 activity in the cecal content of germ-free mice (GFM) and gnotobiotic mice colonized with the gut microbiota of a healthy subject, we bring the proof of concept that a significant DPP-4-like activity occurs in the microbiota. By analyzing the existing literature, we propose that DPP-4-like activity encoded by the intestinal microbiome could constitute a novel mechanism to modulate protein digestion as well as host metabolism and behavior.

## DPP-4 activity of human cells

Initially described in the year 1966, the lymphocyte cell surface protein CD26 presents a proteolytic activity that later on was coined as dipeptidyl peptidase-4 (DPP-4; Hopsu-Havu and Glenner, 1966). Besides on lymphocytes, DPP-4 is found on the surface of a variety of cells, primarily endothelial and epithelial cells, and in the blood in a soluble form (Zhong et al., 2015; Klemann et al., 2016). The proteolytic activity of DPP-4 consists in the cleavage of peptides when proline or alanine is present at the penultimate position of the peptide's N-terminus. The DPP-4 activity takes part in the digestion of dietary proteins (Do et al., 2014) and introduces modifications in bioactive peptides modulating their activity and function (Rosmaninho-Salgado et al., 2012; Cuenco et al., 2017). The listing of potential substrates of DPP-4 activity is broad, ranging from dietary proteins to gut hormones, neuropeptides, and chemokine's (Table 1). Alterations in DPP-4 activity or expression are found in metabolic, intestinal and behavior-related processes, and have led to propose the DPP-4 activity as an essential regulator of different physiological (dys)functions (Elgun et al., 1999; El Yacoubi et al., 2006; Detel et al., 2007; Stengel et al., 2014). Besides, several subsets of immune cells also express on the surface DPP-4, where it plays a signaling role independent on any proteolytic activity (Klemann et al., 2016).

**Table 1:** Selection of some of the substrates of dipeptidyl peptidase-4 (DPP-4) activity [adapted from (Klemann et al., 2016)].

**Peptide**		**N-terminus**	**Biological effect**
Glucagon family	GLP-1	HA↓EG↓TF…	Inactivation
	GIP	YA↓EGTF…	Inactivation
	GLP-2	HA↓DG↓SF…	Inactivation
	GHRH	YA↓DAIF	Inactivation
Neuropeptides	Enterostatin	VP↓DP↓R	Inactivation
	Substance P	RP↓KP↓Q…	Inactivation
	GRP	VP↓LP↓AG…	Inactivation
	β-casomorphin	YP↓FVEPI	Inactivation
	Endomorphin-1	YP↓FF-NH_2_	Inactivation
	Endomorphin-2	YP↓WF-NH_2_	Change in receptor preference
	Morphiceptin	YP↓FP-NH_2_	Inactivation
Pancreatic polypeptides	PYY	YP↓IKPE…	Change in receptor preference
	NPY	YP↓SKPD…	Change in receptor preference
Chemokines	RANTES (CCL5)	SP↓YSSD…	Change in receptor preference
	Eotaxin (CCL11)	GP↓ASVP…	Inactivation
	MDC (CCL22)	GP↓YG↓AN	Change in receptor preference
	MIG (CXCL9)	TP↓VVRK	Inactivation
	IP-10 (CXCL10)	VP↓LSRT	Inactivation
	I-TAC (CXCL11)	FP↓MFKR	Inactivation
	SDF-1α	KP↓VSLS…	Inactivation

*GHRH, growth hormone releasing hormone; GIP, gastric inhibitory polypeptide; GLP, glucagon-like peptide; GRP, gastrin-releasing peptide; IP-10, interferon gamma-induced protein 10; I-TAC, interferon-inducible T-cell α chemoattractant; MDC, macrophage-derived chemokine; MIG, monokine induced by gamma interferon; NPY, neuropeptide Y; PYY, peptide YY; RANTES, regulated upon activation, normal T-cell expressed and secreted; SDF-1, stromal cell-derived factor 1*.

## The gut microbiota exhibits DPP-4-like activity that could be implicated in human health

Intense research has improved our understanding of the roles of DPP-4 activity. However, the studies performed so far have only considered the DPP-4 activity of human cells without taking into account that certain fungi and bacteria, part of the human-associated microbial communities, can also exhibit this enzymatic activity (Wallace et al., 1997; Matos et al., 1998; Varmanen et al., 2000; Goldstein et al., 2001; Sanz and Toldra, 2001; Anastasiou et al., 2002; Glaser et al., 2002; Shibata et al., 2003; Walker et al., 2003; Cooper and Woods, 2009; Stressler et al., 2013; Fteita et al., 2015, 2017; Üstün-Aytekin et al., 2016). Only one study has tackled this issue when describing that the DPP-4 activity of the fungus *Blastomyces dermatitidis*, an opportunistic pathogen that can colonize the lungs, cleaves chemokines with deleterious consequences for local infection (Sterkel et al., 2016). The gut microbiota that permanently colonizes the gastrointestinal tract influences the host metabolism and immune function (Sommer and Bäckhed, 2013; Cani, 2017). The possible roles of DPP-4-like activity exhibited by the gut microbiota itself have never been investigated even if several references describe the existence of DPP-4 activity in *Prevotella* (Wallace et al., 1997; Shibata et al., 2003; Walker et al., 2003; Fteita et al., 2015, 2017), and a counterpart activity to the DPP-4 (called Xaa-Pro dipeptidyl-peptidase or PepX activity) is found in *Lactobacillus, Lactococcus*, and *Streptococcus*. (Matos et al., 1998; Varmanen et al., 2000; Goldstein et al., 2001; Sanz and Toldra, 2001; Anastasiou et al., 2002; Glaser et al., 2002; Stressler et al., 2013; Üstün-Aytekin et al., 2016). Besides, the gut microbiota interacts with nutrients and drugs modulating nutritional or pharmacological therapeutic approaches (Delzenne and Bindels, 2018). As one of the strategies in the management of diabetes consists of the inhibition of the DPP-4 activity, it is particularly interesting to investigate the relevance of the DPP-4 activities produced by the gut microbes. A very recent study conducted in our laboratory has, indeed, shown that the administration of the DPP-4 inhibitor vildagliptin can also inhibit the DPP-4 activity in microbial habitats like the cecal content and feces of mice (Olivares et al., 2018). Here, we hypothesize that DPP-4-like activity produced by the gut microbiota (sum of bacterial DPP-4 plus PepX activities) could be a new mechanism through which the microbes influence physiological or pathological processes in humans. Our hypothesis is based on the key role attributed to DPP-4 in mammals, which could be partly accomplished by the DPP-4-like activity of microbial origin. In the first part of this article, we introduce the features of DPP-4-like activity of microbial origin. Next, we provide the first evidence for DPP-4-like activity produced by the gut microbiota *in vivo*. Finally, we describe how, theoretically, the gut microbial DPP-4-like activity could influence host digestion, metabolism, and behavior.

## Characteristics of DPP-4-like activity in microbes and eukaryotic cells

**Figure 1:**
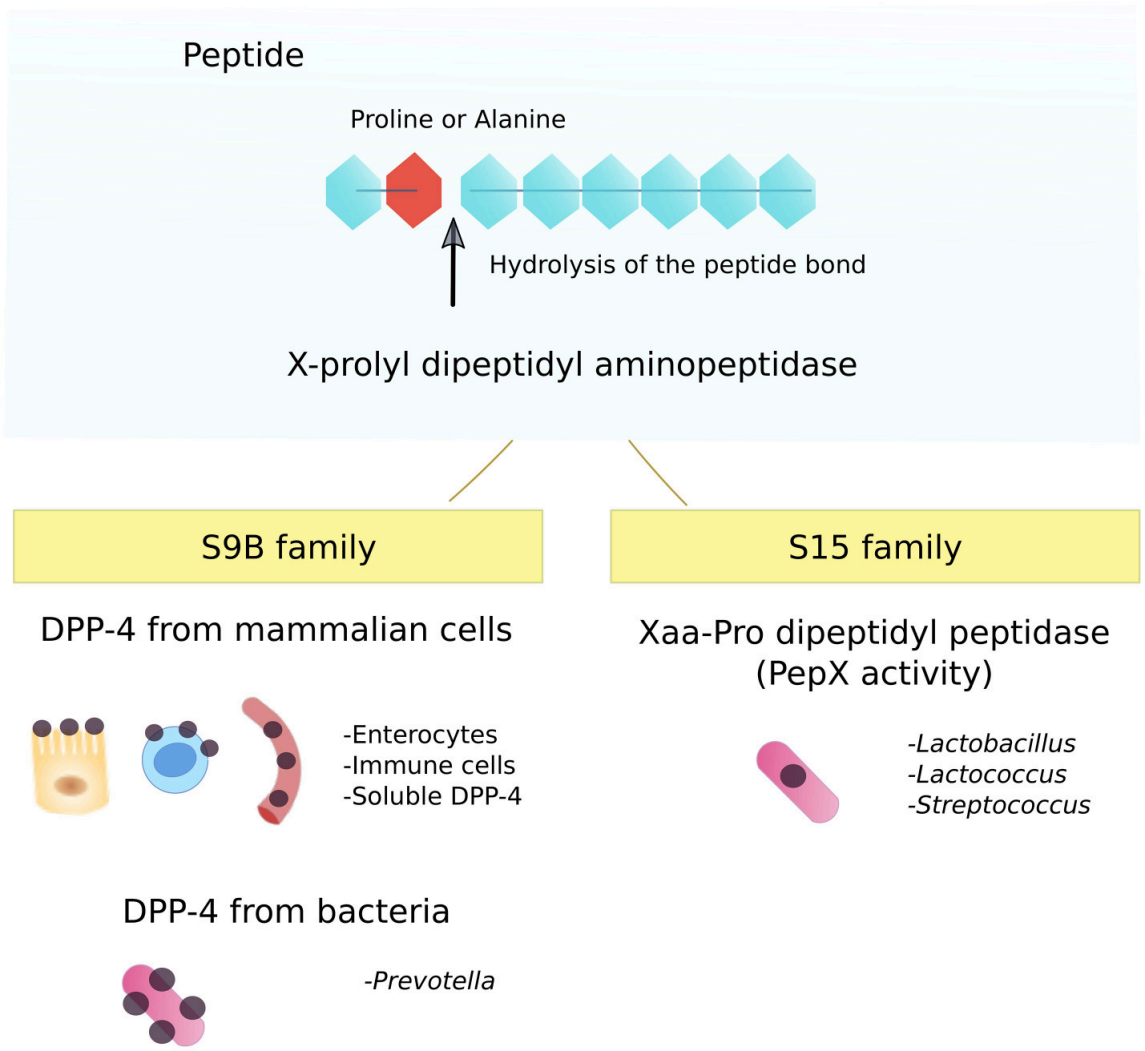
Families with X-prolyl dipeptidyl aminopeptidase activities. The S9B family includes the Dipeptidyl peptidase-4 activity form mammalian cells and Gram-negative bacteria (*Prevotella*). The counterpart activity, so-called Xaa-Pro dipeptidyl peptidase or PepX activity is present in Gram-positive bacteria (*Lactobacillus, Lactococcus, Streptococcus*).

Broadly, all peptidases catalyze the same reaction, the hydrolysis of a peptide bond. However, peptidases do so with a high selectivity. When an enzyme hydrolyzes the N-terminal dipeptide with proline or alanine at the penultimate place, the activity is named X-prolyl dipeptidyl aminopeptidase (Rawlings et al., 2016). There are two families of enzymes that catalyze this reaction: the S9B and S15 peptidase families (Figure 1). Both share the motif GXSYXG as a consensus for the X-prolyl dipeptidyl aminopeptidase activity (Rawlings et al., 2016).

### Family S9B: DPP-4

The typical example of the peptidase family S9B is the DPP-4 from mammalian cells (Rawlings et al., 2016). DPP-4 exists in two isoforms: as a homodimer anchored to the membrane of different cell types (i.e., enterocytes, endothelial or immune cells), and as a soluble circulating form (Zhong et al., 2015; Klemann et al., 2016). When expressed on the surface of mammalian cells, the structure consists of a cytoplasmic region, a transmembrane portion, and an extracellular tail with the catalytic triad (Klemann et al., 2016). The soluble form is thought to be released from the membrane of immune cells to the blood (Gorrell et al., 2001; Cordero et al., 2009). The DPP-4 activity is highly conserved and can also be found in fungi and certain Gram-negative bacteria, like in certain species of the genus *Prevotella* (Wallace et al., 1997; Shibata et al., 2003; Walker et al., 2003; Fteita et al., 2015, 2017). The DPP-4 from eukaryotic and prokaryotic organisms present a very similar amino acid sequences and in both cases, it corresponds to the number 3.4.14.5 according to the Enzyme Commission (Shibata et al., 2003; Walker et al., 2003).

### Family S15: xaa-pro dipeptidyl peptidase or PepX activity

The typical example of the peptidase family S15 is the Xaa-Pro dipeptidyl peptidase from *Lactococcus lactis* coded by the *pepX* gene, and consequently named PepX activity (Rawlings et al., 2016). This can be found in lactic acid bacteria, such as *Lactobacillus, Lactococcus*, and *Streptococcus* (Matos et al., 1998; Varmanen et al., 2000; Goldstein et al., 2001; Sanz and Toldra, 2001; Anastasiou et al., 2002; Glaser et al., 2002; Stressler et al., 2013; Üstün-Aytekin et al., 2016). PepX is a cytosolic enzyme associated with the oligopeptide transporter oppA. OppA introduces oligopeptides into the bacterium that afterwards, are cleaved by the PepX activity (Wang et al., 2012). The PepX and DPP-4 activities are described as counterparts, meaning that both cleave the same peptide bond. However, despite sharing the same selectivity toward the peptide bond, there is no similarity in their amino acid sequence beyond the residues implicated in the enzymatic activity (Walker et al., 2003; Rigolet et al., 2005). According to the Enzyme Commission, the PepX activity corresponds to the entry 3.4.14.11.

## DPP-4 and PepX activities described in bacteria of the gut microbiota

The Table 2 summarizes the bacterial members of the intestinal microbiota with reported DPP-4 or PepX activities. In ruminants, the DPP-4 activity of *Prevotella* is described as one of the enzymes that break down peptides in the rumen (Walker et al., 2003). In the human oral microbiome, the DPP-4 activity of *Prevotella* has been considered as a virulence factor involved in the biofilm formation and the onset of periodontal disease (Fteita et al., 2017). In *Lactobacillus* and *Lactococcus*, the PepX activity has been studied in the field of food technology as it contributes to the hydrolysis of bitter proline-containing peptides influencing texture and flavor of dairy and fermented meat products (Sanz and Toldra, 2001; Gatti et al., 2008). Also, the PepX activity is involved in the generation of bioactive peptides from casein (Stressler et al., 2013). In the genus *Streptococcus*, the *pepX* gene is described as one of the genes required for the infection processes of *Streptococcus gordonii*-responsible for bacterial endocarditis- and *Streptococcus agalactiae*-causative of neonatal sepsis- (Goldstein et al., 2001; Glaser et al., 2002).

**Table 2:** Commensal bacteria of the gut microbiota with reported DPP-4 activity.

**Family**	**Gen**	**Protein**	**ID**	**Bacterial specie**	**References**
S9B	*dpp4*	Dipeptidyl peptidase-4	3.4.14.5	*Prevotella aurantiaca*	Fteita et al., 2015, 2017
				*Prevotella intermedia*	Shibata et al., 2003; Fteita et al., 2015
				*Prevotella nigrescens*	Fteita et al., 2015
				*Prevotella pallens*	Fteita et al., 2015
				*Prevotella albensis*	Walker et al., 2003
				*Prevotella ruminicola*	Wallace et al., 1997
S15	*pepX*	X-prolyl dipeptidyl peptidase or PepX activity	3.4.14.11	*Lactobacillus helveticus*	Stressler et al., 2013
				*Lactobacillus sakei*	Sanz and Toldra, 2001
				*Lactobacillus rhamnosus*	Varmanen et al., 2000
				*Lactococcus lactis*	Matos et al., 1998; Üstün-Aytekin et al., 2016
				*Streptococcus thermophilus*	Anastasiou et al., 2002
				*Streptococcus gordonii*	Goldstein et al., 2001
				*Streptococcus agalactiae*	Glaser et al., 2002

## The intestinal microbiota presents DPP-4-like activity: proof of concept

The consideration of the intestinal microbiota as a source of DPP-4-like activity is an entirely new concept. In order to prove this hypothesis, we have analyzed the activity and expression of DPP-4 in samples of germ-free mice (GFM) and gnotobiotic mice colonized with feces of a lean subject (see Supplemental Material and Methods).

**Figure 2:**
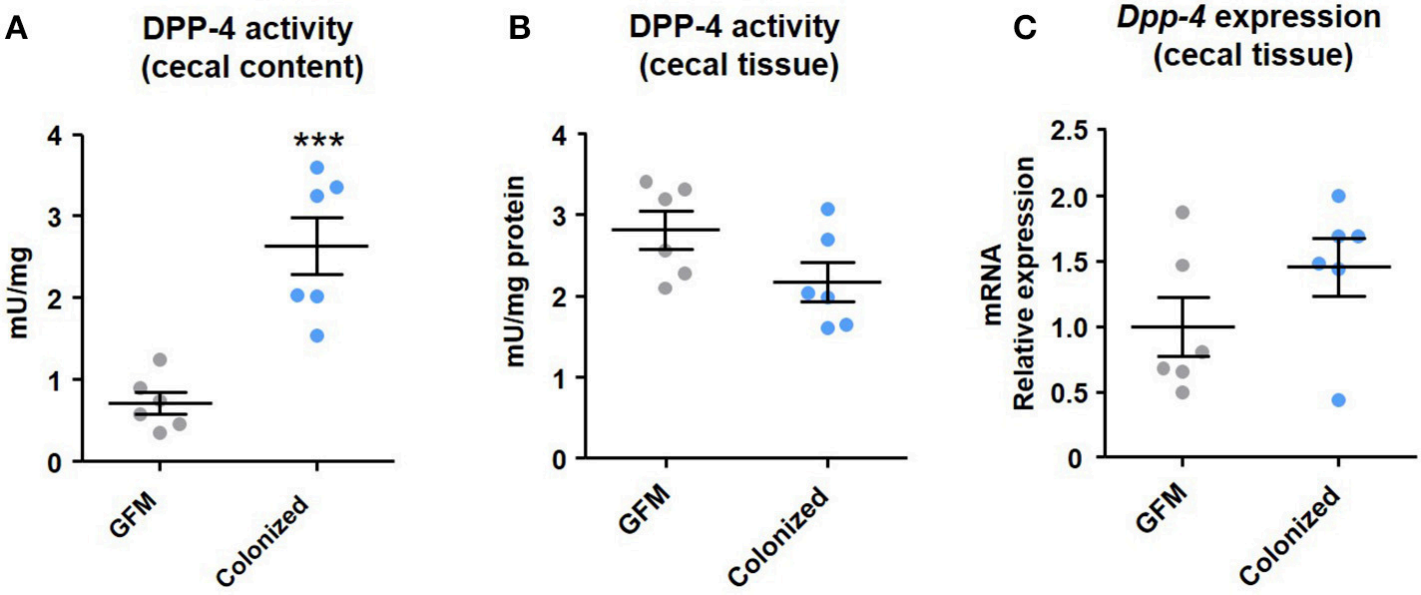
Dipeptidyl peptidase-4 in the **(A)** cecal content and **(B)** cecal tissue, and **(C)** *Dpp4* expression in the cecal tissue of germ-free mice (GFM) and mice colonized with healthy gut microbiota. Data were analyzed with the *t*-test for unpaired samples. Significant differences are represented by the symbol ***(*p* < 0.001).

In GFM, the DPP-4 activity in the cecal content represents the activity that is released from eukaryotic cells. The renewal of enterocytes drives the shedding of intestinal tissue constituents into the lumen (including DPP-4) (Rawlings et al., 2016). In colonized mice, the DPP-4 activity was significantly higher compared to GFM (*p* < 0.001) (Figure 2A). This increase is attributed to the DPP-4-like activity produced by the intestinal microbiota. Our conclusion is reinforced by the absence of differences in the DPP-4 activity and *Dpp-4* expression in the cecal tissue between the GFM and the colonized mice (Figures 2B,C). The slight but not significant reduction of the DPP-4 activity in the cecal tissue of the colonized mice (*p* = 0.087) is in agreement with two previous studies reporting a decrease of the activity in the brush-border of germ-free piglets and rats compared to the colonized equivalents (Kozáková et al., 1998; Kozakova et al., 2006). Conversely in B10.BR/SnPh mice, it has been reported an absence of differences in the DPP-4 activity in the brush-border between GFM, gnotobiotic and conventional mice at 3 weeks-old (Kozáková et al., 2001). However, when the mice were older (8 weeks-old), GFM presented lower DPP-4 activity (Kozáková et al., 2001). A higher cell renewal due to stimulation by the gut microbiota could have increased the DPP-4 activity of colonized mice; however, this hypothesis is not confirmed across studies, and besides it depends on the age of the mice. In the present study, we did not find differences in the DPP-4 activity or expression in the intestinal tissue between GFM and colonized C57Bl6/N mice (16 weeks-old). Thus, we can state that the increase in the DPP-4 activity in the cecal content of colonized mice is produced by the intestinal bacteria.

## DPP-4-like activity of the gut microbiota: potential impact on host physiology and health?

**Figure 3:**
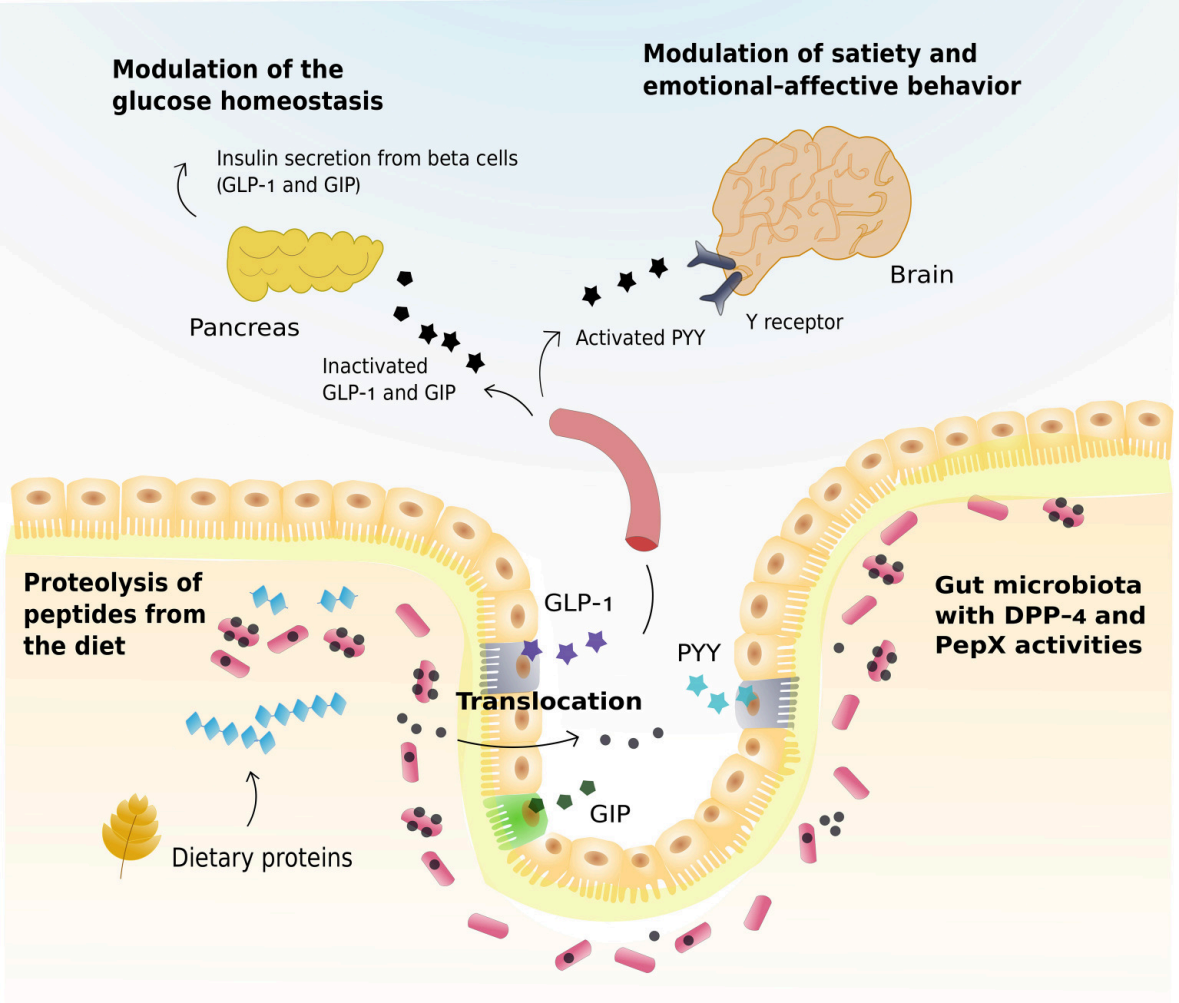
Theoretical scenarios in which the Dipeptidyl peptidase-4 and PepX activities produced by the gut microbiota might impact host's health through the proteolysis of different substrates with a biological activity. Glucagon-like peptide-1 (GLP)-1, gastric inhibitory polypeptide (GIP), and peptide YY (PYY) are represented in color (purple, green and blue, respectively) when not cleaved by DPP-4 and PepX activities. The corresponding symbols are represented in black after the cleavage by DPP-4 and PepX activities.

The DPP-4 and PepX activities produced by the gut microbiota might influence host physiological functions if they can cleave substrates with a biological activity, like dietary peptides, gut hormones or neuropeptides. We elaborated different scenarios in which the DPP-4 and PepX activities of the gut microbiota might impact nutrient digestion and host metabolism and behavior, in view of the existing literature (Figure 3).

### Digestion of proteins

During the digestion, protein and oligopeptides are hydrolyzed by pancreatic proteases. Smaller peptides are, then, cleaved by brush-border peptidases to produce free amino acids and di- and tripeptides. The DPP-4 activity at the surface of enterocytes is one of the peptidases involved in the last step of protein digestion (Do et al., 2014). The DPP-4 activity is produced all along the intestinal tract, but the activity is higher in the ileum than in the caecum or colon (Olivares et al., 2018). As mentioned above, the DPP-4 activity results in the release of a dipeptide if proline or alanine is present at the penultimate position. Interestingly, human gastrointestinal proteases are unable to degrade dietary proteins with high proline content, and then, we hypothesize that enzymatic activities of the intestinal bacteria, including the DPP-4 or PepX activities, can contribute to their digestion (Shan et al., 2005). The interest of the proteolytic activity of the gut microbiota has been demonstrated in food intolerances triggered by gluten, a group of proteins characterized by a high proline content, but these studies were not particularly focused on the DPP-4 or PepX activities (Laparra and Sanz, 2010; Caminero et al., 2016). The role of the PepX activity in the breakdown of gluten has been studied, for instance, to obtain sourdough free of gluten (De Angelis et al., 2010). More specifically, fungal proteases from *Aspergillus oryzae* and *Aspergillus niger* can release polypeptides from gluten, which are subsequently transported inside lactic acid bacteria and hydrolyzed (De Angelis et al., 2010). In this process at least three peptidases, the PepX activity, the aminopeptidase type N (PepN), and endopeptidase O (PepO), are necessary to detoxify the immunogenic epitopes of gluten in the sourdough (De Angelis et al., 2010).

On the other hand, a high proteolytic activity of the intestinal microbiota has long been regarded as detrimental since some bacterial metabolites produced from amino acid degradation (i.e., H_2_S, ammonia, and phenol) are toxic for the epithelium (Blachier et al., 2007). However, more recent data have shown that other compounds produced by intestinal bacteria from amino acids, such as indole, are protective for the mucosa (Bansal et al., 2010). The consequences of an increase in bacterial proteolytic activity for the host are difficult to predict and, thus, the exact significance of the DPP-4-like activity of the microbiota as a player in the cleavage of dietary proteins warrants to be investigated.

### Host metabolism

The role of DPP-4 activity in the control of glucose metabolism is the one most extensively studied (Zhong et al., 2015). After nutrient ingestion, enteroendocrine cells (L and K cells) release gut hormones, like incretins, into the bloodstream. The two main incretins, glucagon-like peptide 1 (GLP-1) and gastric inhibitory polypeptide (GIP) are among the list of substrates of the DPP-4 activity (Seino et al., 2010) (Table 1). As a result, GLP-1 and GIP present a short half-life because the DPP-4 activity of hematopoietic and endothelial cells rapidly inactivates them (Mulvihill et al., 2017). Consequently, *Dpp-4* knockout (KO) mice showed an improved glucose tolerance associated with increases in the GLP-1 levels (Marguet et al., 2000). In humans, this effect is achieved by the administration of DPP-4 inhibitors, a type of antidiabetic agents that preserve the active form of incretins (Zhong et al., 2015).

Strong evidence corroborates a link between the gut microbiota and glucose homeostasis (Delzenne et al., 2015). In that sense, several approaches have aimed to handle the gut microbiota to promote the release and activity of intestinal hormones. For instance, the stimulation of the production of GLP-1 through the administration of prebiotic nutrients (that change the commensal gut microbiota composition and activity), has proven efficacy for improving glucose metabolism and satiety in humans and animal models (Delzenne et al., 2011; Everard and Cani, 2014). Also, the use of commensal bacteria, as *Lactobacillus*, able to release GLP-1 analogs has been proposed as an innovative way to improve host functions related to energy and glucose metabolism (Lin et al., 2016). As a fresh perspective, could the DPP-4 and PepX activities produced by the gut microbiota inactivate GLP-1 and GIP, thereby impairing glucose homeostasis as a consequence? For this purpose, DPP-4 and PepX activities would need to be released and cross the gut barrier to interact with the gut hormones. Mice KO for *Dpp-4* gene and fed with a control diet exhibit in the plasma a residual DPP-4 enzymatic activity (~10% compared to the wild-type mice) whose origin is unknown (Marguet et al., 2000). This activity could, at least in part, come from the translocation of DPP-4 activity from the microbiota and that even if the amount of enzyme were small, it may have an effect on the host's metabolism. Accordingly, for instance, previous human studies have reported that overweight and obese subjects in comparison to normal-weight subjects present an increase in the DPP-4 activity of 2.7 and 13.5%, that is associated with decreases in active GLP-1 of 22.5 and 27.7%, respectively (Ahmed et al., 2017). Also, animal studies performed in our group have shown that the administration of prebiotics causes a drop of the DPP-4 activity of the 23.1% that is associated with a double concentration in the active form of GLP-1 (Cani et al., 2005). So, previous data seems to point out that small changes in the DPP-4 activity can be associated large changes in the active form of GLP-1. However, the translocation of active enzymes from the gut microbiota to host organs or tissues, or the inactivation of GLP-1 and GIP by activities different from host enzymes has never been demonstrated and remains hypothetical, as well as the connection between the increases in *Prevotella* in metabolic disorders and the DPP-4 activity of a bacterial origin (Pedersen et al., 2016). Some recent evidence shows the translocation of a bacterial protein (i.e., caseinolytic protease Clp B produced by *E. coli*) in the intestine that mimics the action of α-melanocyte-stimulating hormone and controls pathways of host satiety (Breton et al., 2016). In the case of the DPP-4, much remains to be investigated to unravel if the DPP-4 activity produced by the gut microbiota could play any role in the host metabolism.

### Satiety and emotional-affective behavior

The communication that is established between the gut and the brain is known as the gut-brain axis. Among the molecules involved in this dynamic exchange of information are some gut hormones, like the neuropeptide Y (NPY) and peptide YY (PYY) (Holzer et al., 2012). PYY is exclusively expressed by endocrine cells of the digestive system, whereas NPY is found in several cell systems at distinct levels of the gut-brain axis (for instance, enteric and sympathetic neurons; Holzer et al., 2012). DPP-4 also modulates the functionality of NPY and PYY (Table 1). DPP-4 can cleave off the N-terminal dipeptide enhancing the agonistic activity of the shortened PYY (PYY_3−36_) at Y2 receptors. In this way, the effect of PYY in the ileal and colonic brake of digestion and in the induction of satiety via activation of hypothalamic Y2 receptors can be enhanced (Cox, 2007; Holzer et al., 2012). However, the role of DPP-4 in the regulation of satiety has not yet been fully sorted out. Thus, DPP-4 deactivates GLP-1 but enhances the activity of NPY, and PYY at Y2 receptors. While *Dpp-4* KO mice are protected against obesity (Conarello et al., 2003), DPP-4 inhibitors lack an effect on body weight in humans (Karagiannis et al., 2012).

There is some evidence indicating that DPP-4 is also involved in the regulation of emotional-affective behavior. *Dpp-4* KO mice exhibit an anti-depressive and hyperactive phenotype (El Yacoubi et al., 2006). Likewise, DPP-4-deficient congenic rats showed enhanced stress resilience (Canneva et al., 2015). The DPP-4 inhibitor sitagliptin has an antidepressant effect as it reduces the immobility time in the forced swim test and tail suspension test in mice, and prevents the high fat diet-induced prolongation of immobility time in the forced swim test in rats (Kamble et al., 2016; Magdy et al., 2017). In humans, incretin-based therapies, which include DPP-4 inhibitors and GLP-1 analogs, reduce depression symptoms in patients who have type 2 diabetes. This antidepressant effect of incretins was independent of their effect on hemoglobin A1C (Moulton et al., 2016). The mechanism of the antidepressant effect of DPP-4 inhibition remains unclear but could be related to changes in the functionality of GLP-1, NPY, and PYY, given that enhancement of GLP-1 and Y1 receptor signaling has an antidepressant action (Redrobe et al., 2002; Isacson et al., 2011; Anderberg et al., 2016). In rodents, DPP-4 inhibitors reduce neuronal loss and cognitive impairment in ischemic stroke, an effect which is independent of the hypoglycemic action of DPP-4 (Shannon, 2013; Ma et al., 2015). It is worth noting that DPP-4 inhibitors do not cross the blood-brain barrier, which implies that the neuroprotective effect of DPP-4 inhibitors is mediated by a peripheral route rather than by central inhibition of DPP-4 (Shannon, 2013). DPP-4-like of bacterial origin has never been assessed as a potential modulator of food related-behavior, mood or depression. Once again, this must be evaluated taking into account the complexity of the processes linking the gut to the brain functions.

### DPP-4 in gut inflammation and visceral pain

As DPP-4 plays a substantial role in the immune system, particularly in T cell function, DPP-4 has been investigated as a possible target for treating autoimmune diseases including inflammatory bowel disease (Klemann et al., 2016). While circulating DPP-4 activity and the amount of the enzyme in plasma and colon is reduced in patients with active Crohn's disease, DPP-4 positive lymphocytes are higher in patients compared to healthy controls (Hildebrandt et al., 2001; Moran et al., 2012). Importantly, the effects of pharmacological DPP-4 inhibition and *Dpp4* KO on intestinal inflammation are not consistent with each other. While systemic and topical DPP-4 inhibitors reduce colitis severity and promote healing of colitis (Mimura et al., 2013; Salaga et al., 2017), *Dpp4* KO does not protect from colitis (Geier et al., 2005) but increases colonic myeloperoxidase activity and nuclear factor κbp65 subunit and modifies leucocyte trafficking (Detel et al., 2012). The substrates of DPP-4 include several pain-modifying mediators as endomorphin-2 and substance P (Table 1). Accordingly, *Dpp4* KO in mice promoted increases of the substance P concentrations in the plasma but not in the brain (Guieu et al., 2006). This effect is associated with reduced latency in the hot plate and tail pinch tests, a change that reflects mechanical and thermal hyperalgesia (Guieu et al., 2006). In clinical practice, DPP-4 inhibitors occasionally lead to arthritis and arthralgia (Mascolo et al., 2016). As regards visceral pain, several DPP-4 substrates are involved in the regulation of visceral nociception. For example, the GLP-1 analog liraglutide reduces visceral hypersensitivity induced by lipopolysaccharide injection and water avoidance stress in rats (Nozu et al., 2018). ROSE-010, another GLP-1 analog, reduces visceral pain in patients suffering from irritable bowel syndrome (Hellström et al., 2009). The KO of *Pyy* or *Y1* receptors in mice and the pharmacological blockade of peripheral Y2 receptors, increase sensitivity to visceral pain which suggests an involvement of PYY and other Y1 and Y2 receptor ligands in the regulation of visceral pain sensitivity (Naveilhan et al., 2001; Hassan et al., 2017). Any involvement of microbial DPP-4-like in the bioavailability of gut hormones regulating visceral pain sensitivity and colitis remains to be explored.

## Conclusion

The host-microbiota cross-talk involves interactions with dietary factors and mediators of the neuroendocrine and immune systems. Here we provide new evidence of the ability of gut microbiota to produce DPP-4-like activity by comparing GFM and colonized mice. We describe the different contexts in which microbial DPP-4 activity could be interesting to evaluate. The functions of the DPP-4-like homolog expressed in the gut microbiota could influence dietary protein digestion and thereby contribute to the change in the host response toward these peptides. The influence on the modulation of host endocrine peptides, which play crucial roles in the metabolism and behavior, are more hypothetical given the lack of data reporting the potential translocation of DPP-4-like from the gut lumen to host tissues. Future experiments are needed to investigate this hypothetical mechanism and potential quantitative contribution of these bacterial activities. This hypothesis paper suggests another way by which the gut microbiota may contribute to nutrients metabolism, and thereby affect host physiology. If confirmed in humans, it will significantly contribute to the progress in our understanding of the links between the human microbiome and human diseases.

## Data availability statement

The data that support the findings of this study are available from the corresponding author upon justified request.

## Ethics statement

Mouse experiments were performed with permission of the German animal welfare authorities at the district government (Regierung von Oberbayern, reference number 55.2-1-54-2532-27-14).

## Author contributions

MO, AH, MB, PH, and ND drafted the original manuscript. MO prepared the figures. VS and DH performed the animal experiment. MO acquired the data and analyzed the data. All authors (MO, VS, AH, MB, AN, LB, AB-P, YS, DH, PH, and ND) provided intellectual input, contributed to discussion and approval of the final manuscript. ND supervised the manuscript preparation and is the guarantor of this work.

### Conflict of interest statement

The authors declare that the research was conducted in the absence of any commercial or financial relationships that could be construed as a potential conflict of interest.

## Abbreviations

DPP-4: Dipeptidyl peptidase-4
GFM: germ-free mice
GLP: Glucagon-like peptide
GIP: Gastric inhibitory polypeptide
KO: Knockout
PYY: Peptide YY
NPY: Neuropeptide Y.
