# Supplementary material for: The Potential Role of the Dipeptidyl Peptidase-4-Like Activity From the Gut Microbiota on the Host Health
Source: Front Microbiol. 2018 Aug 22;9:1900. doi: 10.3389/fmicb.2018.01900 (PMC6113382; doi:10.3389/fmicb.2018.01900)
Supplement: Supplementary file 1 [file Data_Sheet_1.docx]

**Supplemental Material and Methods**

**Ethics Statement for Animal Studies and Housing Conditions**

Mouse experiments were performed with permission of the German animal welfare authorities at the district government (Regierung von Oberbayern, reference number 55.2-1-54-2532-27-14). All mice were housed in open cages in groups of three to four animals within flexible film isolators ventilated via HEPA-filtered air with constant 12-hour light/dark cycle (Kleintierforschungszentrum TU Munich, Freising, Germany). Sterility of germ free mice (GFM) at the beginning of the experiment (and also at the end of the experiment for GFM) was controlled by plating feces on agar plates and gram stainings. A mold trap was used to check the presence of mold. Mice had free access to sterile water and purified control diet (S5745-E902, Ssniff, Soest, Germany). Colonization efficiency of colonized mice was tested by plating dilution series of feces every two weeks.

**Preparation of fecal bacterial suspension and cecal tissue/content sampling**

Six GFM (male, C57Bl6/N) were gavaged at the age of four weeks with a single inoculum of the fecal bacterial suspension obtained from a lean human donor as previously described (1). Briefly, 400 mg of frozen pulverized human fecal aliquot was thawed on ice and then dissolved in 2 ml sterile reduced PBS (PBS supplemented with 0.05% L-cysteine-HCl) in an anaerobic chamber followed by 5 min vortexing and 5 min sedimentation. Sedimentated supernatant was transferred to pre-gassed hungates for colonization procedure. At the age of 16 weeks, colonized mice (n=6) and a group of age-matched GF mice (n=6), used as the control group, were sacrificed by CO2 inhalation. The cecal contents and cecal tissues were taken sterile and stored at -80°C until analysed.

**Cecal tissue and content sampling**

Six GF male C57Bl6/N mice were gavaged at the age of four weeks with a single inoculum of the bacterial supernatant obtained from a lean human donor as previously described [1]. Briefly, 400 mg of each pulverized human fecal aliquot was dissolved in 2 ml sterile reduced PBS (PBS supplemented with 0.05% L-cysteine-HCl) in an anaerobic chamber followed by 5 min vortexing and 5 min sedimentation. Supernatant was transferred to pre-gassed hungates for colonization procedure. At the age of 16 weeks, colonized mice (n=6) and a group of age-matched GF mice (n=6), used as the control group, were sacrificed by CO_2_ inhalation. The cecal content and cecal tissue were taken sterile and stored at -80°C until analysed.

**Measurement of DPP-4 activity**

The DPP-4 activity was measured through the cleavage of para-nitroanilide (PNA) from the substrate Gly-Pro-PNA. Briefly, 20-50 mg of the samples (cecal content or cecal tissue) were suspended in Tris based buffer (50 mM, 1% of n-octyl-glucoside, pH 8.3) and homogenised for 2 min with a tissue lyser. Samples were centrifuged (3,000 g, 20 min). 20 µL of the supernatant was incubated with the substrate Gly-Pro-PNA. The enzymatic activity was measured in a kinetic of 30 minutes at 37°C with absorbance measurements (380 nm) every minute (SpectraMax M2, Molecular Devices). The DPP-4 activity was quantified with a standard curve of PNA. In the cecal content, the results were expressed as mU/g. In the cecal tissue, the values were normalized relative to the amount of tissue protein quantified by the Bradford method and the activity expressed as mU/mg protein.

**Gene expression analyses**

Total RNA was isolated from tissues using the TriPure isolation reagent kit (Roche Diagnostics, Germany). Complementary DNA was prepared by reverse transcription of 1 µg of total RNA using the kit Reverse Transcription System (Promega, Madison, WI, USA). Real-time PCR was performed with the StepOne System (Applied Biosystems, The Netherlands). Samples were run in duplicate and the data were analyzed using the 2^-ΔΔCT^ method. The expression of the targeted gene was normalized with the expression of the ribosomal protein L19 (*Rpl19*). The sequences used were: *Rpl19*-F: GAA GGT CAA AGG GAA TGT GTT C; *Rpl19*-R: CCT TGT CTG CCT TCA GCT TGT. *Dpp4*-F: CAC TGC AGTA CCC CAA GAC A; *Dpp4*-R: GAG CCG CAC TAG AGG ATG AG.

**Statistical analysis**

Statistical analysis was performed the *t*-test for unpaired samples using the GraphPad Prism Version 5.00 software (San Diego, CA, USA). The results were considered statistically significant at *p*<0.05.

[1] Ridaura VK, Faith JJ, Rey FE, et al. (2013) Gut microbiota from twins discordant for obesity modulate metabolism in mice. Science 341: 1241214
